# Integrating Near-Infrared Spectroscopy and Proteomics for Semen Quality Biosensing

**DOI:** 10.3390/bios15070456

**Published:** 2025-07-15

**Authors:** Notsile H. Dlamini, Mariana Santos-Rivera, Carrie K. Vance-Kouba, Olga Pechanova, Tibor Pechan, Jean M. Feugang

**Affiliations:** 1 Department of Animal and Dairy Sciences, Mississippi State University, Starkville, MS 39762, USA; 2 Department of Biochemistry, Nutrition, and Health Promotion, Mississippi State University, Starkville, MS 39762, USA; 3 Institute of Genomics, Biocomputing, and Bioinformatics, Mississippi State University, Starkville, MS 39762, USA

**Keywords:** biomarkers, biosensing, extracellular vesicles, male fertility, NIRS, semen quality, seminal plasma, proteomics, pig

## Abstract

Artificial insemination (AI) is a key breeding technique in the swine industry; however, the lack of reliable biomarkers for semen quality limits its effectiveness. Seminal plasma (SP) contains extracellular vesicles (EVs) that present a promising, non-invasive biomarker for semen quality. This study explores the biochemical profiles of boar SP to assess semen quality through near-infrared spectroscopy (NIRS) and proteomics of SP-EVs. Fresh semen from mature Duroc boars was evaluated based on sperm motility, classifying samples as Passed (≥70%) or Failed (<70%). NIRS analysis identified distinct variations in water structures at specific wavelengths (C1, C5, C12 nm), achieving high accuracy (92.2%), sensitivity (94.2%), and specificity (90.3%) through PCA-LDA. Proteomic analysis of SP-EVs revealed 218 proteins in Passed and 238 in Failed samples. Nexin-1 and seminal plasma protein pB1 were upregulated in Passed samples, while LGALS3BP was downregulated. The functional analysis highlighted pathways associated with single fertilization, filament organization, and glutathione metabolism in Passed samples. Integrating NIRS with SP-EV proteomics provides a robust approach to non-invasive assessment of semen quality. These findings suggest that SP-EVs could serve as effective biosensors for rapid semen quality assessment, enabling better boar semen selection and enhancing AI practices in swine breeding.

## 1. Introduction

Artificial insemination (AI) is one of the most utilized breeding tools in commercial swine programs, offering numerous advantages in genetic improvement, disease control, and reproductive efficiency [1]. Most AI centers rely on fresh, high-quality semen and follow established criteria for processing ejaculates based on genetic merit, aiming to meet defined standards for sperm motility and morphology, usually set at a minimum of 70% [2]. Therefore, semen that does not meet this pre-screening requirement is discarded (Failed), causing substantial economic losses.

To address this issue, alternative research avenues are being explored, with a particular focus on seminal plasma, the non-cellular liquid fraction of semen. Seminal plasma (SP) is a complex mixture of secretions from the testes, epididymis, and male accessory sex glands [3]. SP comprises various bioactive molecules such as proteins, lipids, nucleic acids, and antioxidants, playing crucial roles in modulating sperm functionality [4], specifically motility and morphology [5]. Numerous studies have examined the seminal plasma of various species as a valuable bioactive source for identifying non-invasive biomarkers associated with sperm fertility [6,7,8,9,10]. The complexity of semen is intriguing for various analyses, and the synergistic applications of holistic (i.e., near-infrared spectroscopy or NIRS) and high-throughput (i.e., proteomics) techniques can provide more insights into its biological relevance.

NIRS is a rapid, non-invasive, and non-destructive high-energy vibrational spectroscopy for analyzing the chemical composition of compounds or solutions [11]. It measures the absorption of near-infrared radiation within the 750–2500 nm wavelength range based on interactions with organic functional groups [12]. It has been effectively utilized in examining biological fluids, leveraging the strong overtone signals from the water matrix that are linked to hydrogen bonding, revealing a complex and dynamically changing microstructure [13]. The combination of NIRS with aquaphotomics further explores the water spectrum that arises from interactions between water and specific biological components, shedding light on how material composition influences the formation of water coordination spheres, clusters, and ions, exhibiting specific absorbance patterns described by the water matrix coordinates (WAMACS) [14]. Current research in our laboratory demonstrates NIRS’s ability to classify various types of extended boar semen, making this combined approach a valuable tool for discerning disparities in molecular structures and interactions within boar seminal plasma samples related to semen quality, particularly in terms of sperm motility and morphology.

Proteomics can further enrich NIRS data by providing insights into specific protein expression levels. As spermatozoa mature in the epididymis, they interact with testicular proteins and extracellular vesicles (EVs) found in seminal plasma, which can serve to develop biosensors for selecting semen [15]. Notably, EVs are membrane-enclosed phospholipid nanostructures, categorized as exosomes (40–120 nm) and microvesicles (50–1000 nm), that carry bioactive molecules, including proteins and nucleic acids [16], whose contents mirror that of their parent cells. Interestingly, the EVs found in the seminal plasma (SP-EVs) may influence sperm motility and fertility [17]. However, the biological effects of SP-EVs and their protein contents on sperm function, particularly concerning different quality statuses, are not yet fully understood.

Our limited understanding of the protein composition in boar seminal plasma extracellular vesicles (SP-EVs) with varying quality statuses (Passed vs. Failed) highlights the need for methods to distinguish semen quality phenotypes. This study evaluated the effectiveness of near-infrared spectroscopy (NIRS) in assessing the molecular composition of boar seminal plasma and identified the proteomic content of SP-EVs related to sperm quality. Identifying biomarkers will enable the development of new biosensing techniques for rapid assessment of semen quality, ultimately improving fertility success.

## 2. Materials and Methods

### 2.1. Ethics Statement

Ethical approval was not needed for this study, as the animals were in a commercial boar stud and only semen was donated.

### 2.2. Semen Collection and Sample Preparation

A total of sixty-four fresh semen samples were collected from healthy, sexually mature Duroc boars (n = 64) aged between 1.5 and 2 years. All boars were housed under controlled environmental conditions at a commercial boar farm (Prestage Farms; West Point, MS, USA). The temperature was maintained between 18 °C and 22 °C, with a relative humidity of approximately 52%. The experiments were conducted on a biweekly basis over eight weeks.

### 2.3. Sperm Motion and Morphology Analyses

Aliquots of sperm-rich semen were incubated for 15 min at 37 °C and then subjected to sperm motility and morphology analyses using Computer-Assisted Sperm Analysis (CASA; CEROS II, IVM Biotechnologies, Maple Grove, MN, USA), following previously established protocols [18]. A total of 31 Failed and 33 Passed sperm aliquots were analyzed in triplicate (three fields per chamber), with approximately 300 ± 3 (mean ± SEM) spermatozoa per chamber. The analysis recorded proportions of morphologically abnormal, motile, and progressive spermatozoa, along with various kinetic parameters according to the following CASA preset values: 60 frames/s; frame rate: 60 Hz; motility threshold: 5 μm/s; average path velocity and straightness of progressive cells: 45 μm/s and 45%, respectively; VAP and VSL cut-offs for slow cells at 20 and 5 μm/s, respectively; magnification: 1.89X; temperature: 37 °C. Semen samples were classified as Passed (high-quality) if they contained more than 70% normal morphology and motile spermatozoa. Conversely, those with less than 70% normal morphology and motile spermatozoa were categorized as Failed (low-quality). This classification was determined immediately on-site after collection using established criteria for sperm analysis. Samples classified as Passed were then extended for AI doses. Raw semen samples (50 mL) from both Passed and Failed categories, as well as the corresponding extended doses from the Passed samples, were chilled and transported to the laboratory for processing within one hour of collection. For further analysis, samples from both the Passed and Failed groups were selected based on the mean ± 2 standard deviations of sperm motility and morphology, respectively.

### 2.4. Boar Seminal Plasma Collection

Raw semen (~10 mL) was centrifuged at 800× *g* for 20 min at room temperature to separate sperm cells and seminal plasma gently. The resultant supernatant was centrifuged at 2000× *g* for 20 min at 4 °C to remove residual sperm cells and cell debris. Purified seminal plasma was aliquoted and stored at −80 °C until NIRS analysis and EVs isolation. For the experiments, each aliquot was used only once to minimize the number of freeze-thaw cycles.

### 2.5. NIR Spectral Signature Collection

Transmittance NIRS spectra (n = 64) were obtained using a portable ASD FieldSpec 3+ IndicoPro (Malvern Panalytical, ASD Analytical Spectral Devices Inc., Boulder, CO, USA). SP samples (300 µL) were thawed on ice for 15 min and warmed before NIR spectral acquisition. Each SP sample was placed in a 1.00 mm quartz cuvette positioned within an ASD fiber optic cuvette adapter. NIR spectra were recorded over a wavelength range of 350–2500 nm, with an interval of 1.4 nm for the 350–1000 nm region and 2.0 nm for the 1000–2500 nm region, accumulating 50 scans with a 34 ms integration time. Only wavelength regions within the near-infrared range (1300–1600 nm) were considered as variables for the predictive modeling analysis. Before collecting the plasma spectrum, a reference spectrum was obtained from an empty cuvette. Ten independent spectral signatures were gathered per sample, with the cuvette being repacked with plasma between each replicate. The detector end of the contact probe was sanitized with 70% ethanol between individuals to prevent biochemical interference and cross-contamination. Spectral signatures from the empty cuvette established the baseline for the measurements. Sterile distilled water was utilized as a reference solution and to monitor any dark signal laser drift in the system. Distinct spectral features in the NIR water spectrum characterized the NIR profiles of the sample groups.

#### 2.5.1. Principal Component Analysis

Principal Component Analysis (PCA) and Linear Discriminant Analysis (LDA) were applied to the first overtone region of the near-infrared spectrum, corresponding to the vibrational combination band between 1300 and 1600 nm, using Unscrambler X v.11 (Aspen Technology Inc., Bedford, MA, USA). The PCA-LDA classification model was evaluated using a confusion matrix, which included quality parameters such as accuracy (the percentage of correctly classified samples), sensitivity (the true positive rate), and specificity (the true negative rate) to assess the model’s classification effectiveness.

#### 2.5.2. Aquaphotomics

Aquaphotomics was applied as a complementary spectral analysis to determine the biochemical profile of boar seminal plasma. Principal component analysis was conducted on the mean-centered matrix of the aquaphotomics region (1300–1600 nm) using full random cross-validation and the algorithm SVD (singular value decomposition) to uncover spectral trends [12]. The water microstructure is represented by 12 spectral bands in the first overtone of the OH stretching region, spanning from 1300 to 1600 nm. Water Matrix Coordinates (WAMACS) were used to generate barcodes and aquagrams, enabling the distinction between NIR spectra of Passed and Failed seminal plasma. Barcodes were constructed using normalized absorbance values to determine the peak, followed by the identification of WABS within each of the 12 WAMACS. These points were color-coded by category (Passed or Failed) to facilitate comparison of chemical shifts. For the aquagrams identified at baseline by WABS, a radar chart was plotted to produce WASPS (water absorbance spectral patterns), which reflect the differences in water molecules among the samples. Absorbance normalization was performed using Microsoft Excel 365 by subtracting the mean transformed absorbance of distilled sterile water, which served as the reference solution for each SP group, and then dividing the results by the standard deviation (SD) of each category.

### 2.6. Isolation of Seminal Plasma EVs

A subset of extreme SP samples (*n* = 18) from the Passed and Failed semen groups was selected for proteomic analyses with average sperm motility (88.5 ± 2.0% and 52.2 ± 3.1%) and normal morphology (86.6 ± 1.8% and 48.1 ± 3.9%) for Passed and Failed semen, respectively. To isolate SP-EVs, the boar sperm-rich fraction was first centrifuged at 800× *g* for 20 min at room temperature to separate sperm cells and seminal plasma. The supernatant was removed for additional centrifugation at 2000× *g* for 20 min to remove cell debris and residual sperm cells. This was followed by centrifugation at 16,000× *g* for 1 h at 4 °C to remove residual cell debris. The resulting supernatant (seminal plasma) was ultracentrifuged at 120,000× *g* for 70 min at 4 °C to isolate EVs and the EV-depleted SP fraction. Visible EV pellets were rinsed twice with 5 mL highly purified cold phosphate-buffered saline (PBS) by ultracentrifugation at 120,000× *g* for 70 min at 4 °C. Cleaned EV pellets were resuspended in 100 µL cold PBS aliquots and stored at −80 °C for further analyses.

### 2.7. Characterization of Isolated SP-EVs

#### 2.7.1. Transmission Electron Microscopy (TEM)

The morphology and size of the SP-EVs were analyzed using a transmission electron microscope according to the methods previously reported [19]. Briefly, a drop of five µL of purified EVs was placed on parafilm. The EV drops were covered with formvar carbon-coated electron microscopy grids and incubated for 20 min at room temperature to absorb the EVs. The grids were stained and fixed (with a drop of 2% uranyl acetate for 5 min and 2% methyl cellulose) for 10 min on ice to facilitate drying. The presence of the EVs was examined under an electron microscope TEM-JEOL 2100 (JEOL Ltd., Tokyo, Japan) at 200 kV TEM.

#### 2.7.2. Nanoparticle Tracking Analysis (NTA) of Isolated SP-EVs

Nanoparticle tracking analysis was performed using the ViewSizer 3000 Nanoparticle Tracking Analyzer following the manufacturer’s protocol (Horiba, Irvine, CA, USA) to measure the concentration and size distribution of SP-EVs. Briefly, 25 μL of the EV pellet was diluted 3000× in PBS. Samples were inserted into the cell and placed in ViewSizer 3000. Blue laser power was set to 210 mW and gain to 30. Fifty videos were recorded and processed with Polydisperse settings.

### 2.8. Seminal Plasma EVs Labeling and Indirect Labeling of Spermatozoa

Passed and Failed boar SP-EVs were labeled with a green fluorochrome lipophilic (EX01 ExoSparkler Mem Dye-Green, Dojindo Laboratories, Rockville, MD, USA), following the manufacturer’s recommendations. Briefly, 10 × 10^8^ SP-EVs in PBS (100 μL) were mixed with DMSO-diluted Mem Dye-Green (2 μL) and incubated for 30 min at 37 °C. The labeled SP-EVs were filtered and centrifuged twice to wash with PBS (3000× *g* for 5 min) at room temperature, aliquoted (50 μL), and stored at −80 °C until use. Freshly collected spermatozoa (10^7^) were resuspended in 500 μL PBS, mixed with 50 μL labeled SP-EVs of comparable amounts, and incubated at 37 °C with 5% CO_2_ for 1 and 2 h. Spermatozoa incubated without SP-EVs served as controls. Following incubation, aliquots of labeled spermatozoa were subjected to CASA analysis. Spermatozoa resuspended in PBS were washed through three successive centrifugations to eliminate unbound SP-EVs. The experiment was replicated twice using high-quality semen (sperm motility > 70%).

All samples (labeled SP-EVs, supernatants of each centrifugation, and corresponding washed spermatozoa) were subjected to In Vivo Imaging System (IVIS Lumina XRMS Series III, Waltman, MA, USA). Thereafter, aliquots were fixed in 4% paraformaldehyde, spread on microscope slides, and mounted with a medium containing DAPI to counter-stain the sperm nuclei. The slides were visualized with a Laser Scanning Confocal microscope 710 (Zeiss, Oberkochen, Germany) with a plan-apochromat 63x/1.40 Oil DIC M27 objective, and images were analyzed with the ZEN 2012 SP1 software (blue edition).

### 2.9. Protein Analyses

#### 2.9.1. SP-EV Sample Preparation for NanoLC-MS/MS Analysis

Proteins were eluted from IP beads, and the sample buffer was exchanged into 25 mM ammonium bicarbonate, pH 8.0, as a volatile buffer to improve sensitivity. Protein mixtures were reduced with 10 mM DTT, alkylated with 20 mM iodoacetamide, and digested overnight with trypsin. NanoLC was carried out using a Thermo Scientific UltiMate 3000 (Milford, MA, USA). Mobile phase solvents A and B were 0.1% TFA or Trifluoroacetic acid (*v*/*v*) in water and 0.1% TFA (*v*/*v*) in 95% acetonitrile, respectively. Tryptic peptides were loaded into a μ-Precolumn Cartridge (5 μm, 100 A, 300 μm i.d.) and separated by a Nano LC column (3 μm, 100 A, 75 μm i.d.; Michrom, CA, USA) to elute peptides (0.5 µL/min). The tryptic peptides were separated on an acetonitrile gradient (ranging from 5% to 60%) and fractions were collected at 20 s intervals (2 µL/min flow), followed by Mass Spectrometry analysis on the AB SCIEX TOF/TOF 5800 System (AB SCIEX, Marlborough, MA, USA). Mass spectra (800 to 4000 *m*/*z*) were acquired in reflectron positive ion mode. Tandem MSMS fragmentation spectra were acquired for each ion, averaging 4000 laser shots per fragmentation spectrum (excluding trypsin autolytic peptides and other known background ions). The resulting peptide mass and the associated fragmentation spectra, showing a higher intensity of the precursor ion, were submitted to the MASCOT search engine (Matrix Science, London, UK) to search the Swiss-Prot database. Searches were performed without constraining protein molecular weight or isoelectric point, with variable carbamidomethylation of cysteine and oxidation of methionine residues, and a maximum of one missed cleavage allowed in the search parameters. Matched proteins with Ion C.I.% greater than 95, with a false discovery rate (FDR) of 1%, were considered as identified hits. The relative abundance of proteins in each sample was reflected by the exponentially modified protein abundance index (emPAI) score.

#### 2.9.2. Sperm Immunoprotein Detection

Western immunoblotting was used to confirm the presence of LGALS3BP in frozen-thawed spermatozoa of Passed and Failed semen groups. Isolated EVs were lysed with complete RIPA lysis buffer, and total protein was collected after centrifugation (12,000× *g* for 30 min) at 4 °C and quantified. Total protein (20 μg) of Passed and Failed spermatozoa were separated on 4–12% Tris-Glycine gels and transferred onto PVDF membranes, subjected to western immunoblotting (WesternBreeze Chromogenic Detection kit; anti-rabbit, Thermo Fisher Scientific, Carlsbad, CA, USA), as previously described [20]. In situ fluorescence of both proteins was examined on frozen-thawed spermatozoa derived from Passed and Failed semen groups. Spermatozoa were fixed in 4% paraformaldehyde in PBS (pH 7.4) for 24 h, smeared onto histology microscope slides, air-dried, and subjected to in situ immunofluorescence detection as previously described [21,22]. Primary antibody (LGALS3BP Rabbit pAb, Cat No. A12005, ABclonal Technology, Woburn, MA, USA) was used at 1:500 and 1:100 dilutions for WIB and IF, respectively. For IF, the FITC-tagged secondary antibody (mouse anti-rabbit IgG-FITC, sc-2359, Santa Cruz Biotechnology Inc., Dallas, TX, USA) was used at a 1:200 dilution. The slides were then mounted with DAPI-containing medium (DABCO; Sigma Aldrich, St. Louis, MO, USA) and covered with a coverslip using nail polish. The slides were placed on a Laser Scanning Confocal microscope 710 (Zeiss, Oberkochen, Germany) for visualization.

#### 2.9.3. Bioinformatics Analysis

All identified proteins in SP-EV samples were submitted to the DAVID database (https://davidbioinformatics.nih.gov/; accessed on 4 February 2025) using Uniprot accession numbers to determine their functional classification through Gene Ontology (GO) and Kyoto Encyclopedia of Genes and Genomes (KEGG) pathway enrichment analyses. A *p*-value < 0.05 was considered statistically significant. Protein–protein interaction (PPI) networks for differentially expressed proteins were generated using the STRING database (http://string-db.org/; accessed on 19 February 2025) version 12.0.

### 2.10. Statistical Analysis

Data analysis was performed using SPSS for Windows, version 29.0 (SPSS Inc., IBM; Chicago, IL, USA). Semen groups (Failed and Passed) were analyzed for normality using the Shapiro-Wilk’s test, and group comparisons were performed using a two-way repeated measures Analysis of Variance (ANOVA). Significance was set at a *p*-value ≤ 0.05. The results are expressed as mean ± SEM.

## 3. Results

### 3.1. Sperm Quality Evaluation

Table 1 represents the sperm characteristics between Passed and Failed boar semen. Total sperm motility and morphology in Passed semen (87.5% ± 0.9; 84.0% ± 1.1) were significantly increased (*p* < 0.001) in comparison to Failed semen (56.3% ± 4.3; 53.2% ± 2.4). There was a significant increase in proximal droplet for Failed sperm (*p* ≤ 0.019), while no significant difference was observed for distal droplet (*p* ≥ 0.156), coiled (*p* ≥ 0.071), and bent tail (*p* ≥ 0.057). For the velocity parameters, the average path velocity (*p* ≤ 0.045) and curvilinear velocity were significantly high in Passed sperm (*p* ≤ 0.026). No significant differences were observed in motion parameters (linearity—*p* ≥ 0.705 and straightness—*p* ≥ 0.414), amplitude head (ALH) *p* ≥ 0.169, and beat cross frequency (BCF) *p* ≥ 0.167 between both semen groups.

### 3.2. NIRS Findings

**Figure 1:**
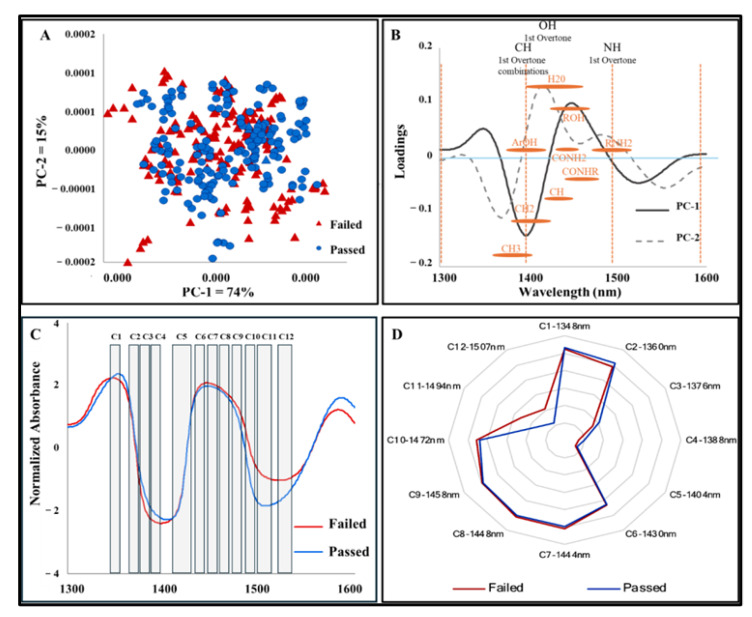
NIR absorbance and aquagram of boar seminal plasma (Passed vs. Failed): (**A**) PCA scores plot for Passed (n = 33) and Failed semen (n = 31); (**B**) PCA loadings showing the dominant peaks influencing the positive and negative trends in the scores plot: PC-1 = 74%, PC-2 = 15%; (**C**) Normalized absorbance of porcine seminal plasma; (**D**) Aquagram created with the key WABS from peaks between Passed and Failed SP.

Spectral variations were observed between 1300 and 1600 nm, corresponding to the first overtone of O-H, C-H, and N-H functional groups found in water (H_2_O), alcohols (ROH), phenols (ArOH), simple amides (CONH_2_), amides (CONHR), monoamides (RNH_2_), methylene (CH_2_), and methyl radicals (CH_3_). These molecules reflect the water-rich and complex biochemical composition of boar SP. Principal Component Analysis (PCA) revealed chemical differences, with some samples showing higher or lower expression levels. Although some overlap existed between groups, PCA scores demonstrated separations, with PC-1 and PC-2 explaining 74% and 15% of the variance, respectively (Figure 1A). The PCA loadings revealed dominant peaks that occurred in the positive trends of the score plot at 1446–1450 nm (PC-1) and 1415–1418 nm (PC-2), and the negative trend at 1396–1399 nm (PC-1) and 1371–1374 nm (PC-2) (Figure 1B). Normalized absorbance spectra highlighted chemical compositional differences between the Failed and Passed seminal plasma groups within the 1300–1600 nm range, especially between the ranges of 1470–1500 nm and 1570–1580 nm, corresponding to the first overtone of N-H stretching vibration (Figure 1C). The WASPS aquagram showed distinct water absorbance patterns at C1, C5, and C12 regions associated with water dimers, bulk water, and kosmotropic solutes, respectively, indicating structural water differences between Passed and Failed SP (Figure 1D).

**Figure 2:**
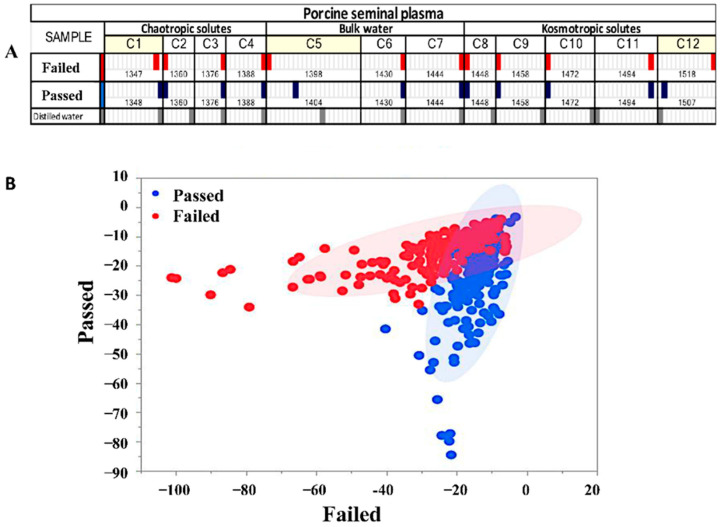
Aquaphotomics of boar seminal plasma (Passed vs. Failed): (**A**) WAMACS barcode showing chemical shifts in Failed (red) and Passed (dark blue) groups compared to distilled water as the reference; (**B**) PCA-LDA plot for the calibration of Model 4 developed with the transformed absorbance (1300–1600 nm) from boar SP of Passed and Failed semen.

The WAMACS barcode distinguished the samples, showing spectral shifts in water matrix coordinates C1, C5, and C12 (Figure 2A). Passed SP samples showed right-shifted peaks at C1 (1398 nm, 1348 nm) and C5 (1398 nm, 1404 nm), indicating a stronger association with bulk water and structured water clusters. Failed samples exhibited a peak at 1518 nm in C12, compared to 1507 nm in Passed samples, suggesting a shift toward chaotropic solutes that disrupt water structure and potentially contribute to protein denaturation.

### 3.3. Discriminant Analysis

A total of 15 PCs were used, accounting for 99.99% of the spectral variance in the database. SP samples had high accuracy, sensitivity, and specificity percentages of 92.2%, 94.2%, and 90.3%, respectively. The PCA-LDA plots showed similarities in the chemical compositions of SP samples (Passed and Failed). However, differences in ratios of volatile and non-volatile compounds in SP samples were identified, indicating biochemical differences in the composition of porcine SP that can subsequently influence sperm quality (Figure 2B).

### 3.4. Morphological and Molecular Characterization of SP-EVs

#### 3.4.1. TEM and NTA Measurements of SP-EVs

**Figure 3:**
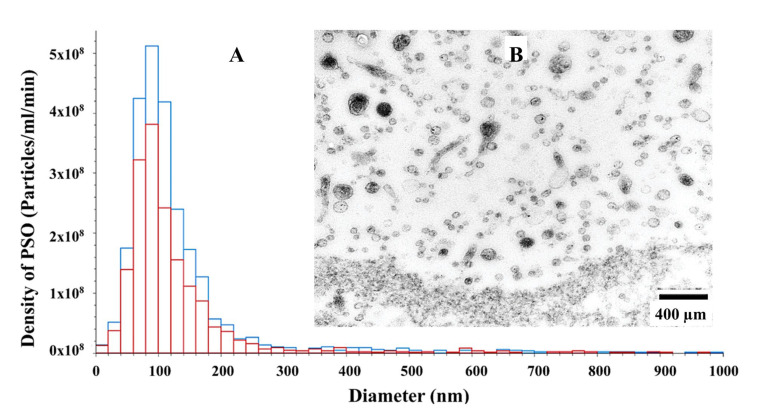
Morphological and molecular characterization of boar SP-EVs: (**A**) nanoparticle tracking analysis of Passed (blue bar) and Failed (red bar) SP-EVs particle size and concentration; (**B**) High-Resolution Transmission Electron Microscopy (HR-TEM) image showing a clear morphology of SP-EVs. The scale bar represents 400 nm.

The results are summarized in Figure 3. There was a significant difference in particle concentrations (*p* = 0.027) between Passed (Blue bars: 4.25 × 10^10^ ± 2.76 × 10^9^ particles/mL) and Failed (Red bars: 2.7 × 10^10^ ± 3.8 × 10^9^ particles/mL) SP-EV samples (Figure 3A). However, the average size (mean ± SEM) of Passed (103.91 ± 0.76 nm) and Failed (102.98 ± 3.14 nm) SP-EVs were comparable (*p* = 0.788). The TEM showed round and spherical SP-EVs with bilipid membranes (Figure 3B).

#### 3.4.2. Evaluation of Co-Incubation of SP-EVs with Boar Spermatozoa

**Figure 4:**
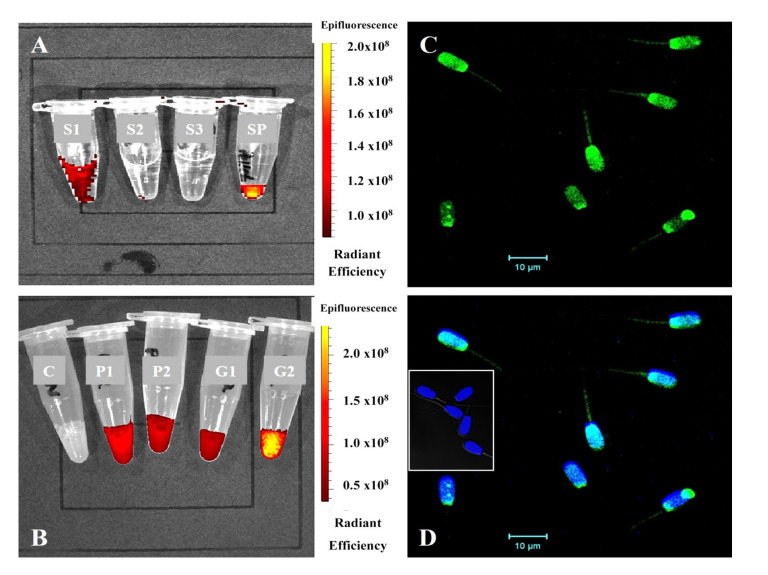
Sperm co-incubation with labeled SP-EVs: (**A**) labeled SP-EVs with spermatozoa after centrifugation and the resultant sperm pellet (S1: Supernatant 1; S2: Supernatant 2; S3: Supernatant 3 and SP: Sperm Pellet); (**B**) labeled SP-EVs with spermatozoa (C: Control; P1: Failed SP-EV 1; P2: Failed SP-EV 2; G1: Passed SP-EV 1 and G2: Passed SP-EV 2); (**C**) confocal microscope imaging of spermatozoa taken two hours after co-incubation with labeled SP-EVs (green staining); (**D**) the sperm nuclei were counterstained with DAPI (blue staining)—The insert indicates control spermatozoa showing no sign of green fluorescence after incubation with or without unlabeled SP-EVs. The scale bar represents 10 μm.

The observed fluorescence indicates the successful labeling of SP-EVs. The progressive removal of the excess dye, as seen in supernatants (S1, S2, and S3) during centrifugations, revealed the fluorescence of the resulting sperm pellet (Figure 4A). Spermatozoa interacted with the SP-EVs, irrespective of their origin, to emit fluorescence signals that were not observed in the control group (Figure 4B). The microscopy imaging confirmed the binding of SP-EVs mainly on the head and mid-piece of spermatozoa (Figure 4C,D).

Table 2 summarizes the impact of SP-EV labeling on the motility characteristics of spermatozoa. At 0-h, total motility, progressive motility, and normal morphology were comparable across all treatment groups, with no significant differences observed (*p* > 0.05). However, total sperm motility significantly decreased during the incubation period at 1 and 2 h, irrespective of the experimental group (*p* < 0.05). The rates of progressive motility for Control, Passed, and Failed SP-EV-treated spermatozoa remained stable at both the 1-h and 2-h intervals (*p* > 0.05). Likewise, normal morphology did not differ between the two groups of SP-EV-treated spermatozoa (*p* > 0.05). Notably, the proportion of progressive motility in SP-EV-treated spermatozoa was not significantly affected after 2 h of incubation, as both progressive motility and normal morphology maintained similar proportions to those observed at 0 h.

### 3.5. Proteome of SP-EVs and Differential Expressions

Supplementary files S1–S3 summarize all generated and curated data of 30 May 2024. Approximately 387 total proteins were detected in both groups of samples, corresponding to 218 and 238 proteins in the SP-EVs of the Passed and Failed semen groups, respectively. Both groups shared 69 proteins (Supplementary file S1). Additionally, nine proteins from the top 20 most abundant, representing approximately 46% of the total expression in each sample group, were shared between both groups. These shared proteins included spermadhesin PSP-1, epididymal boar protein, milk fat globule-EGF factor, and cytoskeletal beta-actin and lactotransferrin. Fibrinogen was the most abundant protein in the Failed group, uniquely possessing proteins such as RAB22A, ubiquitin, and gamma-glutamyl transpeptidase. In contrast, AWN-1 was the most abundant protein in the Passed group, characterized by proteins such as dipeptidyl peptidase, lactoferrin, ezrin, and acrosin (Supplementary file S2). Interestingly, the comparative analysis of the total proteome datasets revealed three differentially expressed proteins (DEPs) between both SP-EV groups (Table 3). Lectin, galactoside-binding, soluble, 3-binding (LGALS3BP) protein was upregulated in Failed SP-EVs, whilst nexin-1 (PN-1) and seminal plasma protein pB1 precursor (BSP1) were upregulated in Passed SP-EVs.

### 3.6. Functional Enrichment Analysis

Over 90% of the detected proteins were successfully converted for GO functional annotation and enrichment analyses (Supplementary file S3). Functional analysis of DEPs in the Failed sample group was associated with enriched GO terms, e.g., regulation of cell shape, cytoplasm, apical plasma membrane, extracellular exosome, and actin filament. In contrast, the Passed sample group revealed numerous enriched GO terms related to extracellular space, extracellular region, cytosol, cell surface, cytoskeleton, single fertilization, protein binding, and intermediate filament organization. KEGG pathway analysis revealed that these proteins were significantly enriched in critical pathways, including the complement and coagulation cascades, glutathione metabolism, and tight junctions, in the Passed sample group. Furthermore, the functional annotation of abundant proteins revealed significantly enriched GO terms associated with single fertilization, secretion, and hydrolase activity.

### 3.7. Immunodetection of LGALS3BP Protein

**Figure 5:**
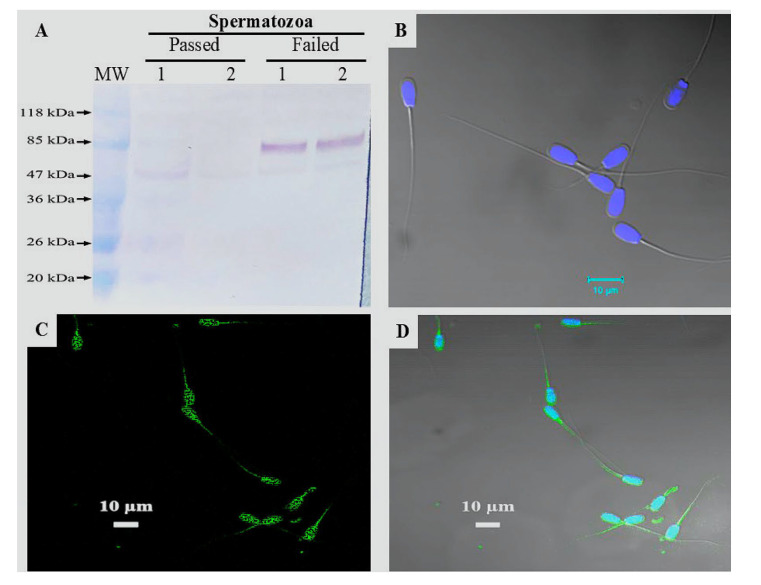
Immunodetection of LGALS3BP in boar spermatozoa. (**A**) Western blotting indicated LGALS3BP protein detection in spermatozoa of Failed semen. Performing immunofluorescence, the negative control corresponded to spermatozoa labeled with a FITC-conjugated antibody in the absence of the anti-LGALS3BP (**B**). Immunofluorescence of LGALS3BP was detected in spermatozoa (FITC green staining—micrograph (**C**). Sperm nuclei are counterstained with DAPI (blue staining—micrograph (**D**)).

Western immunoblotting revealed a strong LGALS3BP expression in Failed spermatozoa (Figure 5). Immunofluorescence detection showed that the protein localization was mainly in the sperm head. Overall, the LGALS3BP signals were lower in spermatozoa of Passed semen. Following the detection and gel-based validation of LGALS3BP in EVs, its presence highlights the potential for developing biosensing platforms.

### 3.8. Protein–to–Protein Interaction (PPI) Analysis

**Figure 6:**
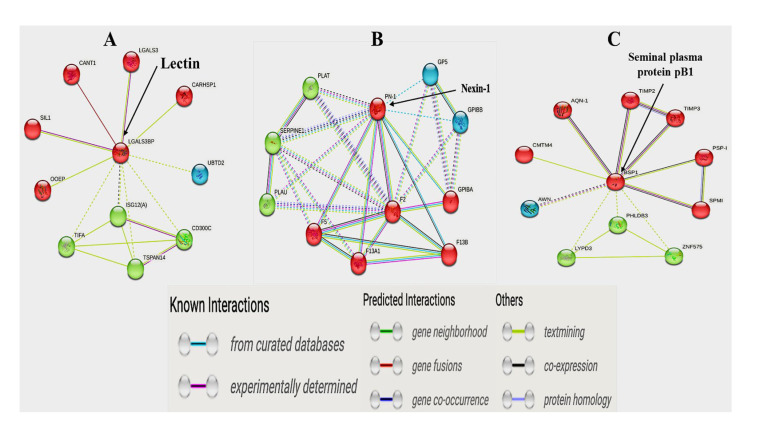
Protein–to–protein interactions (PPI) of differentially expressed SP-EV proteins: (**A**) Lectin (LGALS3BP); (**B**) Nexin-1 (PN-1); (**C**) seminal plasma protein pB1 (BSP1). The proteins are represented by nodes, and different-colored lines connect interacting partners.

To further identify potential key proteins related to sperm quality, we used the STRING database to construct PPI networks of all DE proteins in the Passed and Failed SP-EV samples (Figure 6). LGALSB3P interacted with LGALS3, CANT1, SIL1, OOEP, and CARSP1, amongst others (Figure 6A), which are involved in calcium regulation. On the other hand, nexin-1 primarily interacted with F5, F13A1, F13B, GPIBA, and F2 (Figure 6B), which are associated with blood coagulation. Lastly, seminal plasma pB1 or BSP1 mostly interacted with PSP-1, AQN-1, SPMI, CMTM4, TIMP2, and TIMP3 (Figure 6C), which regulate sperm-egg binding, sperm capacitation, and fertilization.

## 4. Discussion

This study categorized boar semen into two groups—Passed and Failed, based on sperm motility and morphology, which are critical indicators of semen quality and fertility [23]. Semen quality is essential for sperm functionality and fertility outcomes, with motility being particularly important for sperm’s ability to reach and fertilize the oocyte [24]. Our findings confirmed that Passed semen exhibited superior motility and morphology, with fewer sperm abnormalities compared to Failed semen. Failed semen was associated with a higher incidence of sperm with proximal cytoplasmic droplets, indicating incomplete spermatogenesis and impaired sperm maturation, which significantly impacts fertility [25].

Velocity parameters, such as average path velocity (VAP) and curvilinear velocity (VCL), also showed marked differences between Passed and Failed semen. Failed semen exhibited lower VCL and lateral head displacement (ALH), which are directly correlated with reduced fertilization potential in AI and in vitro fertilization (IVF) [26]. These observations highlight the importance of precise semen selection to improve fertility outcomes in swine breeding programs [27].

Near-infrared spectroscopy provided valuable insights into the biochemical composition of SP, offering a non-invasive method for assessing semen quality. When combined with aquaphotomics, NIRS has proven to be a non-invasive method capable of distinguishing the biochemical profiles of various biological fluids, including blood plasma, serum, urine, and milk [27,28,29,30]. In our study, the application of Water Spectral Patterns (WASPS) revealed significant differences in water absorbance bands at the C1, C5, and C12 coordinates, which correspond to water dimers, water clusters in chaotropic solutes (agents that disrupt water structure), bulk water, and kosmotropic solutes (agents that enhance water structure), respectively. These differences suggest shifts in functional groups that reflect chemical alterations linked to semen quality. Our use of NIRS, coupled with PCA-LDA, demonstrated a high degree of accuracy (92.2%), sensitivity (94.2%), and specificity (90.3%) in distinguishing between Passed and Failed samples. Variations in functional groups such as O-H, C-H, and N-H alcohols (ROH), along with various other compounds such as phenols (ArOH), simple amides (CONH2), amides (CONHR), monoamides (RNH2), methylene (CH2), and methyl radicals (CH3) suggest significant chemical shifts in SP related to semen quality. This pioneering study is the first to employ near-infrared spectroscopy for the rapid molecular profiling of boar seminal plasma based on semen quality, paving the way for new advancements in reproductive research.

Beyond NIRS, the study also examined SP-EVs from boar semen. These SP-EVs contain bioactive molecules, such as proteins, lipids, and miRNAs, that influence sperm maturation and function [31,32]. Due to the heterogeneity of their cargo, SP-EVs may serve as non-invasive biomarkers for semen quality and have notable effects post-insemination, which underpin the focus on EVs in this research.

The isolated SP-EVs were characterized with transmission electron microscopy (TEM) and nanoparticle tracking analysis (NTA). The TEM revealed that microvesicles and exosomes have a spherical shape, which is consistent with previous studies [33,34]. Concurrently, NTA indicated that the majority of EV particles are within the 50 to 300 nm size range, aligning with the established characteristics of EVs [35,36].

A uniform distribution of labeled SP-EVs on the sperm head and tail was achieved after a 2-h incubation, in agreement with earlier boar sperm studies exceeding 2 h duration [37,38]. The source, type, and concentration of SP-EVs appear to influence their binding to spermatozoa. Our co-incubation study noted a decrease in total sperm motility after 1 and 2 h compared to unlabeled controls; however, no significant differences in progressive motility or normal morphology were found between the Passed and Failed SP-EV-labeled spermatozoa. These findings suggest that SP-EVs, which carry numerous molecules (i.e., proteins, lipids, sugars, and metabolites), may affect sperm motility and function and influence cells in the female reproductive tract, ultimately impacting embryo development [39,40,41].

The proteomic analysis of SP-EVs revealed distinct molecular signatures between Passed and Failed semen. Notably, proteins such as Nexin-1, BSP1, and LGALS3BP exhibited differential expression patterns. These findings indicate that SP-EVs are not only critical for sperm maturation and capacitation but also possess significant potential for biosensor development aimed at distinguishing between poor-quality or non-viable (Failed) and high-quality or viable (Passed) semen samples. SP-EVs are essential in sperm physiology, affecting motility, acrosome reaction, and fertilization potential [42,43]. Additionally, the proteins identified in this study, including PSP-I, AWN-1, acrosin, and MFG-E8, play crucial roles in sperm membrane stabilization, capacitation, and fertilization processes [44,45]. Pathway enrichment analysis of proteins in the Passed group highlighted significant involvement in glutathione metabolism, tight junctions, and complement and coagulation cascades. These pathways are crucial for oxidative regulation, plasma membrane stability, and immune responses [46,47], adding depth to our findings. In this context, Nexin-1 (PN-1) was found to be upregulated in Passed SP-EVs, whereas LGALS3BP was downregulated in Passed SP-EVs.

LGALS3BP, also known as lectin, galactoside-binding soluble 3-binding protein, belongs to the galectin family, which consists of β-galactoside-binding proteins [48]. The LGALS3BP protein has been identified in various male reproductive tissues, including the testes and epididymal spermatozoa [35]. Research conducted in both humans [49,50] and cats [35] indicated that lectins, such as LGALS3BP, play a role in immunomodulation, sperm capacitation, cellular adhesion, and fertilization potential. In contrast to the findings observed in the cat model, the current study reveals that SP-EVs from failed boar semen contain elevated levels of LGALS3BP protein, which is identified at approximately 80 kDa in spermatozoa and exhibits increased localization in the head region. These contrasting results may highlight the potential for species-specific differences. Investigating LGALS3BP levels in seminal plasma as a prospective biomarker for sperm function and fertility remains critically important, particularly for men undergoing testicular sperm extraction (TESE) [50,51]. Interestingly, LGALS3BP has been demonstrated to play a role in predicting successful sperm retrieval in non-obstructive azoospermia (NOA) patients [49,50,51].

Research indicates that the absence of nexin-1 in both mice and human sperm results in altered semen protein composition, leading to inadequate semen coagulation and seminal dysfunction [52,53]. Additionally, male mice with a deficiency in the gene encoding PN-1 exhibit significant fertility impairments attributed to deficiencies in seminal plasma composition and subsequent alterations in semen protein makeup [53]. Furthermore, SERPINE2, a protease derived from glial cells interacting with PN-1, is involved in serine protease-specific anti-protease activity by inhibiting sperm capacitation [54]. Notably, a higher concentration of SERPINE2 in low-quality sperm has been linked to reduced fertility [55]. These findings support the regulatory role of PN-1 in influencing sperm fertility and seminal function.

Both proteins appear as promising candidates for developing non-invasive diagnostic tools for semen analysis. Research conducted by Durfey et al. [56] and Feugang et al. [57] employed conjugated magnetic and quantum-dot nanoparticles to remove damaged spermatozoa and bio-image boar sperm without affecting sperm quality, showing significant promise for advanced biosensing applications. By tagging these protein candidates (LGALS3BP and PN-1) with fluorochromes or chromogens, it becomes possible to visually distinguish between Passed (viable) and Failed (non-viable) semen samples, including the use of portable NIRS devices for on-farm fertility screening. With a broader utilization in various breeds of boars, this methodological approach could revolutionize the assessment of semen quality in AI and breeding programs.

## 5. Conclusions

This study highlights the promising potential of boar seminal plasma extracellular vesicles (SP-EVs) as biosensors for assessing semen quality. By combining near-infrared spectroscopy (NIRS) with proteomic analysis of SP-EVs, we obtained complementary data enabling rapid, holistic, and reliable differentiation between Passed (viable) and Failed (non-viable) semen. SP-EVs from viable semen contain various proteins that promote sperm functionality, making them effective for viable semen diagnosis. These findings pave the way for more efficient and non-invasive methods for assessing semen quality, utilizing various biosensing approaches to facilitate more precise semen selection and enhance reproductive success.

## Figures and Tables

**Table 1:** Semen characteristics in Passed and Failed boar semen.

Sperm Characteristics	Passed Semen	Failed Semen	*p*-Values
Total motility (%)	87.5 ± 0.9 ^a^	56.3 ± 4.3 ^b^	<0.001
Progressive motility (%)	51.9 ± 3.4 ^a^	36.0 ± 4.4 ^b^	0.002
Normal morphology (%)	84.0 ± 1.1 ^a^	53.2 ± 2.4 ^b^	<0.001
Average path velocity (VAP; μm/s)	74.1 ± 3.3 ^a^	63.3 ± 4.3 ^b^	0.045
Straight-line velocity (VSL; μm/s)	51.0 ± 2.4	44.0 ± 3.4	0.077
Curvilinear velocity (VCL; μm/s)	146.8 ± 6.7 ^a^	124.8 ± 8.3 ^b^	0.026
Bent tail (%)	0.7 ± 0.2	1.8 ± 0.4	0.071
Coiled tail (%)	3.4 ± 0.5	5.3 ± 0.8	0.057
Distal droplet (%)	6.5 ± 0.7	8.1 ± 0.8	0.156
Proximal droplet (%)	16.5 ± 1.7 ^a^	24.6 ± 2.5 ^b^	0.019
Linearity (%)	38.3 ± 1.1	38.8 ± 1.9	0.705
Straightness (%)	71.5 ± 1.2	71.4 ± 1.7	0.414
Amplitude head (ALH; μm)	6.5 ± 0.2	6.1 ± 0.3	0.169
Beat cross frequency (BCF; Hz)	34.8 ± 0.6	33.6 ± 1.0	0.167

Data are mean ± SEM; ^ab^ Means without a common superscript letter in the same line differ significantly (*p* < 0.05).

**Table 2:** In vitro effects of SP-EVs on sperm characteristics. Boar spermatozoa were co-incubated with labeled and unlabeled SP-EVs of Failed (F-EVs) or Passed (P-EVs) semen groups.

Time (h)	Total Motility (%)			Progressive Motility (%)			Norm. Morphology (%)		
	Control	P-EVs	F-EVs	Control	P-EVs	F-EVs	Control	P-EVs	F-EVs
0	65.0 ± 3.8 ^a^	64.5 ± 3.3 ^a^	64.5 ± 3.3 ^a^	26.2 ± 2.5 ^a^	22.4 ± 6.3 ^a^	22.4 ± 6.3 ^a^	86.3 ± 2.0 ^a^	84 ± 4.3 ^a^	84 ± 4.3 ^a^
1	49.1 ± 3.4 ^b^	40.9 ± 7.8 ^b^	36.2 ± 1.7 ^b^	27.5 ± 0.9 ^a^	20.2 ± 6.2 ^a^	19.8 ± 2.8 ^a^	82.8 ± 0.3 ^a^	79 ± 7.1 ^a^	80 ± 0.3 ^a^
2	53.7 ± 0.6 ^ab^	42.6 ± 4.6 ^ab^	41.1 ± 2.4 ^ab^	28.7 ± 1.1 ^a^	30.7 ± 4.4 ^a^	23.5 ± 0.1 ^a^	82.1 ± 1.9 ^a^	85 ± 1.8 ^a^	86.6 ± 1.2 ^a^

^a,b^ Values with different superscripts within columns are significantly different (*p* < 0.05). Norm Morphology = Normal Morphology.

**Table 3:** Differentially expressed boar SP-EV proteins between Passed and Failed SP-EVs identified by nano LC-MS/MS.

Gene Accession No.	Protein Name	Gene ID	*p* Value
SP-EV downregulated proteins (In Passed vs. Failed)			
gi|456752927	Lectin, galactoside-binding, soluble, 3 binding protein	LGALS3BP	<0.001
SP-EV upregulated proteins (In Passed vs. Failed)			
gi|28435507	Nexin-1	PN-1	0.01
gi|3599989	Seminal plasma protein pB1 precursor	BSP1	0.05

## Data Availability

All data are provided in the manuscript.

## Supplementary Materials

The following supporting information can be downloaded at: https://www.mdpi.com/article/10.3390/bios15070456/s1, Supplementary file S1: Total protein detected in Failed and Passed semen SP-EVs. Supplementary file S2: Most abundant proteins in Failed and Passed semen SP-EVs. Supplementary file S3: Functional analysis.

## Abbreviations

The following abbreviations are used in this manuscript:

AI	Artificial insemination
ALH	Amplitude head
BCF	Beat cross frequency
CASA	Computer-assisted sperm analysis
EVs	Extracellular vesicles
GO	Gene ontology
IF	Immunofluorescence
IVF	In vitro fertilization
KEGG	Kyoto encyclopedia of genes and genomes
LC-MS/MS	Liquid chromatography-mass spectrometry
LDA	Linear discriminant analysis
LGALS3BP	Lectin, galactoside-binding, soluble, 3-binding
MFG-E8	Milk fat globule-EGF factor
NIRS	Near infrared spectroscopy
NOA	Non-obstructive azoospermia
NTA	Nanoparticle tracking analysis
PBS	Phosphate-buffered saline
PCA	Principal component analysis
PN-1	Nexin-1
PPI	Protein–to–protein interaction
PVDF	Polyvinylidene difluoride
SD	Standard deviation
SP	Seminal plasma
SVD	Singular value decomposition
TEM	Transmission electron microscopy
TESE	Testicular sperm extraction
VAP	Average path velocity
VCL	Curvilinear velocity
VSL	Straight line velocity
WABS	Water absorbance bands
WAMACS	Water Matrix Coordinates
WASPS	Water absorbance spectral patterns
